# P-1504. Pneumococcal Vaccination Coverage among High-Risk Population in the Unified Health System in Brazil: An Administrative Database Analysis

**DOI:** 10.1093/ofid/ofaf695.1688

**Published:** 2026-01-11

**Authors:** Ricardo Macarini Ferreira, Daniela V Pachito, Rodrigo Alexandre, Paulo Almeida

**Affiliations:** Pfizer Brazil, São Caetano do Sul, Sao Paulo, Brazil; Pfizer Brazil, São Caetano do Sul, Sao Paulo, Brazil; Pfizer Brazil, São Caetano do Sul, Sao Paulo, Brazil; Pfizer Brazil, São Caetano do Sul, Sao Paulo, Brazil

## Abstract

**Background:**

Immunization against pneumococcal disease (PD) for individuals with certain coexisting health conditions is guided by the recommendations of the National Immunization Program (NIP) in the Unified Health System (SUS) in Brazil. There are uncertainties regarding the size of the population at risk of PD in Brazil and the proportion of high-risk individuals that are assessed at the Reference Centers for Special Immunobiologicals (Centros de Referência para Imunobiológicos Especiais, CRIE). Objectives were to assess the high-risk population size by using the outpatient claims database of Brazilian National Health System (SUS), and to estimate vaccination coverage rates.

**Box 1:**
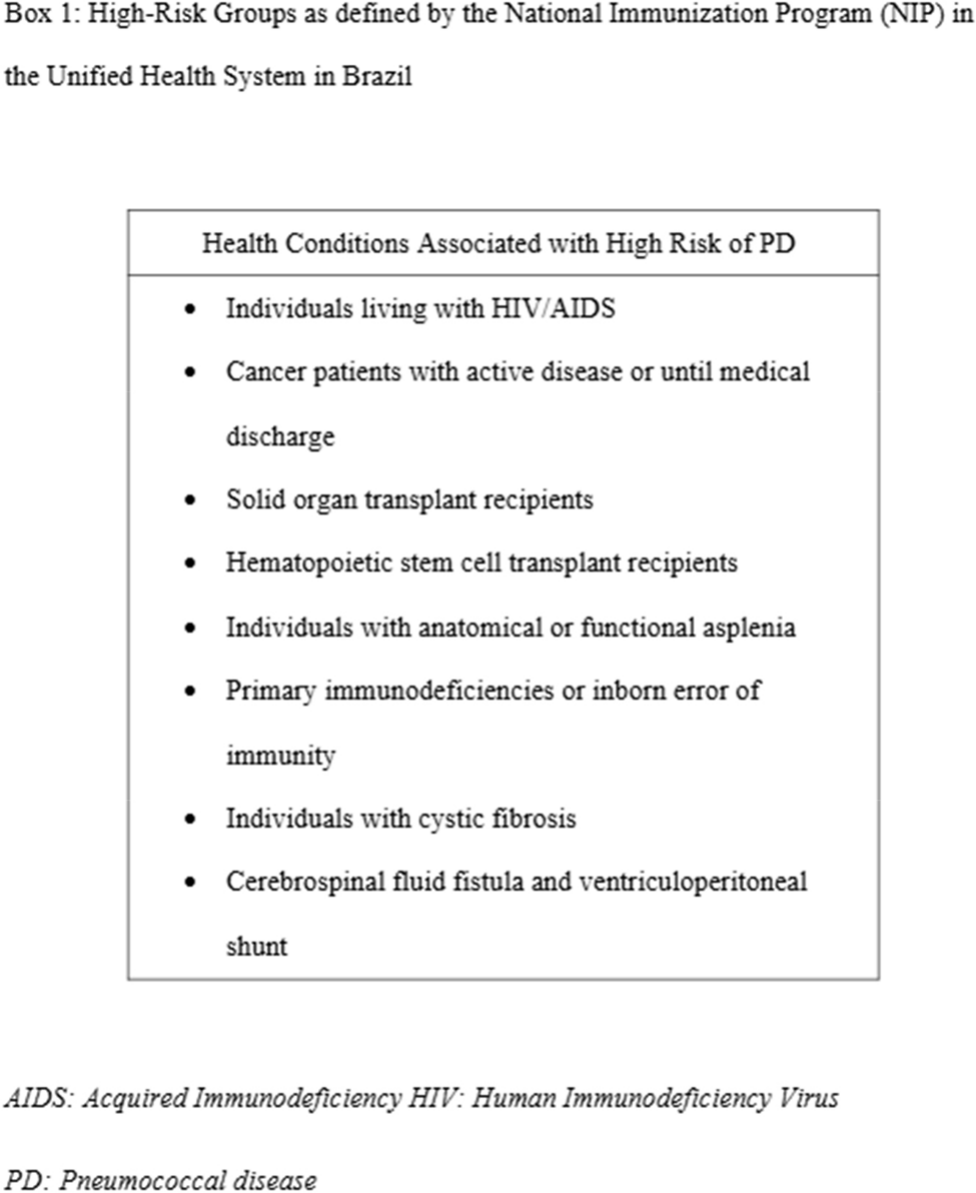
High-Risk Groups as defined by the National Immunization Program (NIP) in the Unified Health System in Brazil

**Figure 1:**
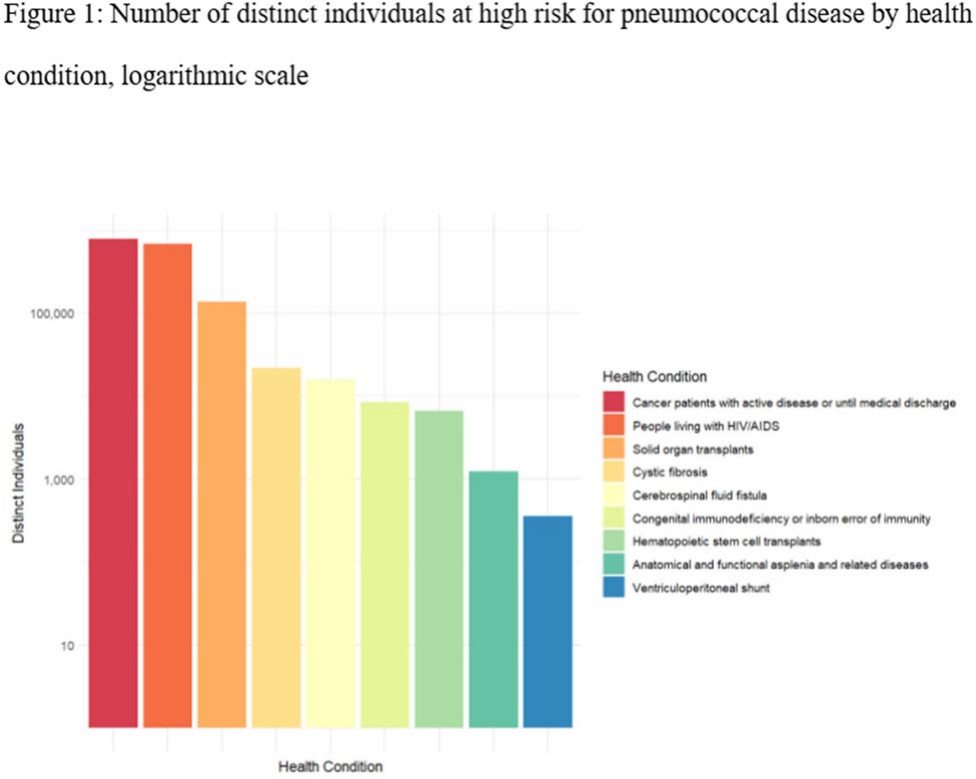
Number of distinct individuals at high risk for pneumococcal disease by health condition, logarithmic scale

**Methods:**

ICD-10 and procedure codes related to high-risk health conditions, as defined by the NIP (Box 1) were defined, together with proxies of disease activity and severity. Data from DATASUS-SIA, the outpatient information system of medium-to-high complexity levels of care in the SUS, and the information system of immunobiologicals (SI-PNI) were analyzed. Encrypted identifiers were used to allow for the counting of distinct individuals from November 2021 to October 2023. Complete vaccination schemes were defined as having received at least one PCV13 and two or more PPS23 doses; incomplete vaccination schemes as one PCV13 and less than two PPS23 doses; and non-conforming vaccination schemes as more than one PCV13 and less than two PPS23 doses.

**Results:**

Estimated size of the high-risk population was 1,609,181 individuals (Figure 1). Coverage rate for having received at least one dose of PCV13 was estimated at 45.5% and for the complete vaccination scheme at 3.5% of the high-risk population. Among vaccinated individuals, most received incomplete (N= 249,132, 34%) or non-conforming vaccination schemes (427,940, 58.4%), while a minority received the complete vaccination scheme (n=55,595, 7.6%) (Table 1).

**Conclusion:**

A small proportion of high-risk individuals was estimated to have received the complete pneumococcal vaccination scheme, possibly due to the high complexity of the vaccination scheme, and limited access to CRIE, considering the continental size of Brazil and the concentration of CRIE in large cities. These findings should be considered in the design of vaccination strategies targeting PD in SUS.

**Disclosures:**

Ricardo Macarini Ferreira, Md/ MBA, Pfizer Brazil: Employee Daniela V. Pachito, MD PhD MBA, Pfizer: Employee|Pfizer: Stocks/Bonds (Private Company) Paulo Almeida, PhD, Pfizer Brazil: Employee

